# Engineering Carrier Dynamics in Halide Perovskites by Dynamical Lattice Distortion

**DOI:** 10.1002/advs.202300386

**Published:** 2023-10-09

**Authors:** Bai‐Qing Zhao, Yulu Li, Xuan‐Yan Chen, Yaoyao Han, Su‐Huai Wei, Kaifeng Wu, Xie Zhang

**Affiliations:** ^1^ Beijing Computational Science Research Center Beijing 100193 China; ^2^ State Key Laboratory of Molecular Reaction Dynamics Dalian Institute of Chemical Physics Chinese Academy of Sciences Dalian Liaoning 116023 China; ^3^ University of Chinese Academy of Sciences Beijing 100049 China; ^4^ School of Materials Science and Engineering Northwestern Polytechnical University Xi'an 710072 China

**Keywords:** carrier dynamics, halide perovskites, lattice engineering

## Abstract

The electronic structure of halide perovskites is central to their carrier dynamics, enabling the excellent optoelectronic performance. However, the experimentally resolved transient absorption spectra exhibit large discrepancies from the commonly computed electronic structure by density functional theory. Using pseudocubic CsPbI_3_ as a prototype example, here, it is unveiled with both ab initio molecular dynamics simulations and transmission electron microscopy that there exists pronounced dynamical lattice distortion in the form of disordered instantaneous octahedral tilting. Rigorous first‐principles calculations reveal that the lattice distortion substantially alters the electronic band structure through renormalizing the band dispersions and the interband transition energies. Most notably, the electron and hole effective masses increase by 65% and 88%, respectively; the transition energy between the two highest valence bands decreases by about one half, agreeing remarkably well with supercontinuum transient‐absorption measurements. This study further demonstrates how the resulting electronic structure modulates various aspects of the carrier dynamics such as carrier transport, hot‐carrier relaxation, Auger recombination, and carrier multiplication in halide perovskites. The insights provide a pathway to engineer carrier transport and relaxation via lattice distortion, enabling the promise to achieve ultrahigh‐efficiency photovoltaic devices.

## Graphical abstract

Electronic band renormalization induced by lattice distortion critically impacts the carrier dynamics in halide perovskites. Here, the origin of a discrepancy is unveiled between the theoretical and experimental electronic band structures, which enables a new pathway to improve carrier transport and relaxation via lattice engineering.

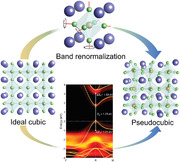

## 1 Introduction

Halide perovskites are exceptional candidates for photovoltaic and light‐emitting devices, with a champion solar conversion efficiency approaching 26%.^[^ ^1^ ^]^ The remarkable optoelectronic performance is intimately related to their microscopic electronic structure, which determines basic properties such as bandgap and effective masses, and derived properties such as absorption, luminescence, carrier mobilities, and carrier recombination rates.^[^ ^2^, ^3^ ^]^

Experimental techniques such as angle‐resolved photoelectron spectroscopy (ARPES),^[^ ^4^, ^5^ ^]^ allow to directly probe the electronic band structure of the valence bands. However, due to complications from sample details and experimental conditions, it could be very challenging to experimentally map the entire band structure. For example, ARPES experiments have led to contradictory results about the giant Rashba splitting in the valence band (VB) of lead‐halide perovskites.^[^ ^6^, ^7^ ^]^ By contrast, first‐principles calculations can overcome this limitation and have been routinely employed to compute the electronic band structures of various materials. In particular, with the developments of accurate hybrid exchange‐correlation functional and many‐body approaches such as GW, the electronic band structure can be theoretically determined precisely.^[^ ^8^ ^]^

The electronic structure of halide perovskites has been extensively studied using first principles.^[^ ^9^, ^10^, ^11^, ^12^, ^13^ ^]^ Reliable approaches based on the Heyd‐Scuseria‐Ernzerhof (HSE)^[^ ^14^ ^]^ hybrid functional^[^ ^15^, ^16^, ^17^ ^]^ or GW^[^ ^18^, ^19^ ^]^ in conjunction with spin‐orbit coupling (SOC) have been established. However, an accurate assessment of the electronic structure is only possible if correct atomic structures for halide perovskites have been employed as input.

Using both theory and experiment for a prototypical halide perovskite CsPbI_3_, we show that the atomic structure of pseudocubic CsPbI_3_ exhibits pronounced dynamical lattice distortion. The pseudocubic phase of CsPbI_3_ at room temperature is only cubic on average; the instantaneous atomic structure contains disordered octahedral tilting. With more realistic atomic structures, we demonstrate that the electronic band structure is substantially altered. The effective masses and interband transition energies reveal giant renormalization, which ultimately affect the carrier dynamics (e.g., transport, relaxation, recombination, and multiplication) in halide perovskites. Our study suggests that lattice distortion can be employed to modulate the carrier dynamics and ultimately the optoelectronic performance.

## 2 Results and Discussion

### 2.1 Discrepancy Between Experiment and Theory

**Figure 1:**
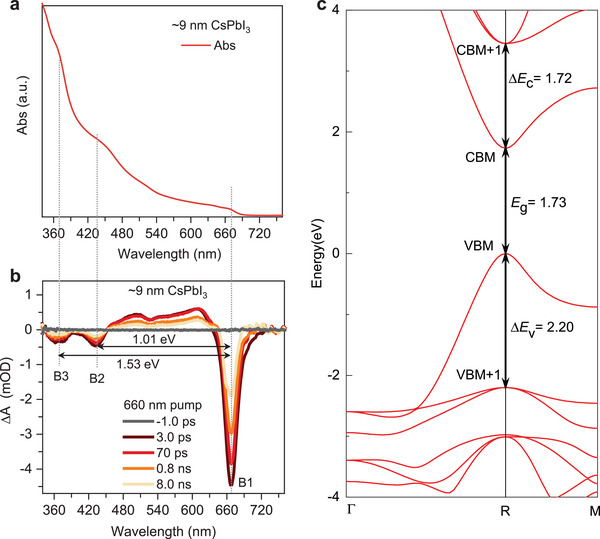
Optical absorption and band structure of CsPbI_3_. a,b) Experimentally measured absorption spectrum (a) and transient absorption spectrum (b) of 9.0 nm CsPbI_3_ nanocrystals dispersed in hexane at room temperature. c) Band structure of cubic CsPbI_3_ computed from first principles with the HSE+SOC scheme.

**Figure** **1**a presents the experimentally measured absorption spectrum of black‐phase CsPbI_3_ nanocrystals (see Experimental Section for synthetic details). The band‐edge absorption peak is at ∼670 nm (or 1.85 eV), which is slightly blue‐shifted compared to that of bulk black‐phase CsPbI_3_ because of quantum confinement as a result of its finite nanocrystal size (∼9 nm). In addition to the band‐edge feature, there exist two extra absorption “bumps” at 440 and 365 nm, which are associated with higher‐energy electronic bands.

The absorption features can be better visualized and assigned using broadband transient absorption (TA) spectra covering ultraviolet to near‐infrared spectral regions. The TA spectra in Figure 1b are acquired with 660 nm pump, which directly address the band‐edge transition. Note that our samples are colloidal dispersions of monodisperse perovskite nanocrystals with size <10 nm, and therefore there exists negligible optical scattering, as proved by the TA spectrum plotted at a negative pump‐probe delay. In addition to the band‐edge bleach feature (labeled as “B1”), the two bleach features associated with the absorption “bumps” above are also present (“B2” and “B3”). Because TA bleach arises from state‐filling effects of photogenerated carriers, simultaneous observation of B2 and B3 bleaches upon exciting into B1 indicates that they share a common state from the conduction‐band minimum (CBM) or valence‐band maximum (VBM) with B1. On the basis of the spin selection rules, a previous study has assigned B3 to a transition from the VBM to the second‐lowest CB (CBM+1), which is split from the CBM by SOC.^[^ ^20^ ^]^ Specifically, when the CsPbI_3_ nanocrystals are excited by a σ^+^ (left‐handed circular polarization) photon resonant with B1, B1 has a maximum bleach under a σ^+^ probe photon but a negligible bleach under a σ^−^ (right‐handed circular polarization) probe photon, and B3 has a maximum bleach under a σ^−^ probe photon. This is consistent with the fact that B3 is dominated by the transition from the VBM (*m*_*h*_ = ±1/2) to the heavy‐electron level in the CBM+1 (*m*_*j*_ = ±3/2) that is separated from CBM (*m*_*j*_ = ±1/2) by the SOC splitting. This assignment is further substantiated by the gap of 1.53 eV between B1 and B3 matching the well‐accepted SOC value of ∼1.5 eV for lead‐halide perovskites.^[^ ^3^, ^21^ ^]^ By contrast, B2 probed in the same experiment shows no spin selectivity. It cannot be associated with a transition from the VBM to a level in the CB, because, at the experimentally observed energy separation of ∼1 eV, there is no level in the CB. This leaves B2 to be likely associated with a transition from the second‐highest VB (VBM+1) to CBM.

The observation of three bleach features is not specific to the nanocrystal size or A‐site (ABX_3_) cation composition. All these features are observed in different‐size CsPbI_3_ nanocrystals as well as in FAPbI_3_ (FA: formamidinium) nanocrystals (see Figure S1, Supporting Information). Crucially, the B1‐B2 and B1‐B3 separations of ∼1.5 and ∼1.0 eV, respectively, are universal to these samples. The studied CsPbI_3_ perovskite nanocrystals are in the size range of 5–9 nm, which is in the intermediate‐to‐weak confinement regime.^[^ ^22^ ^]^ Although the band‐edge transition (B1) indeed blueshifts from ca. 665 to 635 nm with decreasing nanocrystal sizes, the B1‐B2 and B1‐B3 energy separations are less sensitive to the nanocrystal sizes, likely because the CBM and CBM+1 (or VBM and VBM+1) are shifted by the confinement effect in a similar way. Previous reports on MAPbI_3_ (MA: methylammonium) films revealed a similar B1‐B2 separation of ∼1 eV,^[^ ^23^, ^24^ ^]^ although the B3 feature was missing due to limited spectral width of the probe beam in those studies. Above all, these results suggest that B1‐B2 and B1‐B3 separations are intrinsic to lead‐iodide perovskites, and that quantum confinement and A‐site cation have weak impacts on these energies. In principle, accommodating different sizes of A‐site cations should lead to differences in these energy separations, but these differences might be too small to be fully captured by our TA measurements.

The extracted transition energies enable a careful benchmark of the electronic band structure from first principles. Figure 1c shows the band structure of cubic CsPbI_3_ by using accurate HSE+SOC scheme with a fraction of the Fock exchange that allows to reproduce the the experimental bandgap (≈1.73 eV) for bulk CsPbI_3_ thin films at room temperature^[^ ^25^, ^26^ ^]^ (see Experimental Section for details). Although the bandgap is correct, the intra‐VB (Δ*E*_*v*_) and intra‐CB (Δ*E*_*c*_) transition energies are substantially different from the experimental values. With our first‐principles calculations, we obtain 1.72 eV for Δ*E*_*c*_ and 2.20 eV for Δ*E*_*v*_.

### 2.2 Evidence for Dynamical Lattice Distortion

The large discrepancy between theory and experiment in the intra‐VB and intra‐CB transition energies sets an interesting puzzle: what is missing in the first‐principles calculations? The HSE+SOC scheme has, in principle, proven great accuracy and reliability in computing the electronic structure of halide perovskites.^[^ ^27^ ^]^ However, we notice that dynamical lattice distortion has been experimentally reported for halide perovskites at room temperature.^[^ ^28^, ^29^ ^]^ Although often being ignored in previous computations, this lattice distortion might have important consequences for the electronic band structure.

**Figure 2:**
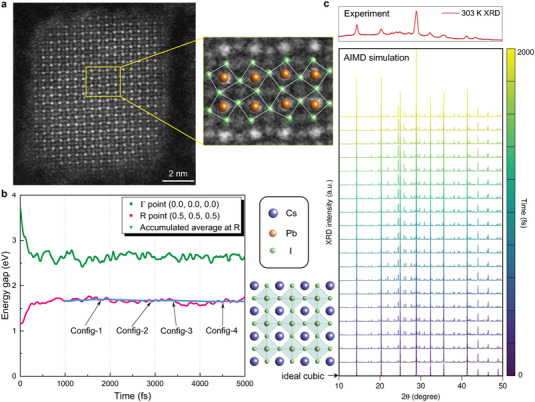
Dynamical lattice distortion in pseudocubic CsPbI_3_. (a) High‐resolution transmission electron microscope image of a typical CsPbI_3_ nanocrystal, exhibiting clear octahedral tilting. (b) Evolution of the energy gaps of CsPbI_3_ at high symmetry points Γ (0.0, 0.0, 0.0) and R (0.5, 0.5, 0.5) as a function of time. The blue line shows the accumulated average value of the bandgap at R (starting from 1000 fs). Four representative configurations (Config‐1 − Config‐4) are selected for more detailed analysis of the electronic structure. The initial atomic structure (ideal cubic perovskite) for the AIMD simulation is depicted. (c) X‐ray diffraction pattern of CsPbI_3_ nanocrystals at 303 K in comparison with those of the configurations extracted from AIMD simulations (from 0 to 2000 fs).

We perform structural characterizations to identify the lattice distortion in the CsPbI_3_ nanocrystals at around room temperature. **Figure** **2**a shows a high‐resolution transmission electron microscope (TEM) image of a typical CsPbI_3_ nanocrystal. The nanocrystal has a cubic shape with an edge‐length of 10 nm, and the overall lattice is pseudocubic‐like. Scrutiny of the image, however, reveals substantial lattice distortion, arising from tilting of Pb‐I octahedra (see the enlarged image in Figure 2a). In principle, Fast Fourier Transform (FFT) analysis of the high‐resolution TEM images may yield quantitative information on the lattice distortion; however, it is infeasible here due to sample instability under electron beams. In our recent study,^[^ ^22^ ^]^ we applied temperature‐dependent X‐ray diffraction (XRD) to quantify this lattice distortion. The orthorhombic lattice constants *b* and *a* were found to increase and decrease with decreasing temperature, respectively, indicative of an enhanced lattice distortion with decreasing temperature. In spite of this lattice distortion, however, the overall crystal framework formed by corner‐sharing PbI_6_ octahedra is still preserved, and the lattice constant changes can be treated as a primarily orthorhombic strain applied to the cubic phase.

We note that the TEM image provides averaged results of the lattice; many instantaneous configurations are averaged during the acquisition time of a few seconds. Thus, these results serve only as a qualitative evaluation of the dynamical lattice distortion. Directly tracking single‐particle lattice distortion on ultrafast timescales is beyond the current experimental capability. In order to extract more realistic atomic structure of CsPbI_3_ in real time, we therefore perform ab initio molecular dynamics (AIMD) simulations of CsPbI_3_ at room temperature. The initial configuration is a 3 ×3 ×3 supercell of the ideal cubic perovskite structure of CsPbI_3_. Using this configuration as the starting point, we can observe and analyze the structural evolution of the cubic phase during the simulation, as well as their impact on the electronic band structure. Figure 2b shows the evolution of the energy gaps at different high‐symmetry **k**‐points (Γ and R) as a function of the AIMD simulation time. The energy gap at Γ quickly decreases and the gap at R (the actual bandgap) gradually increases within the first 1000 fs. This clearly demonstrates that lattice vibrations and distortions at room temperature substantially impact the electronic structure and indicates the instability or lack of relaxation of the initial cubic structure. However, once thermodynamic equilibrium has reached (after ∼1000 fs), the structures is still changing in every step, but the lattice is always on a similar level of distortion from the ideal cubic perovskite (Figure S3, Supporting Information), which leads to similar electronic‐structure renormalization. Notably, the observed behavior is almost independent of the initial structure selected for the simulation. We simulate the X‐ray diffraction (XRD) patterns of the AIMD configurations as depicted in Figure 2c, which can be compared with the experimental result for CsPbI_3_ measured at 303 K (upper panel in Figure 2c). The XRD pattern begins with the one for ideal cubic CsPbI_3_, but very quickly additional diffraction peaks start to develop due to lattice distortions and remain similar after ∼1000 fs. The new sharp XRD peaks are partly due to the finite size of the supercell. However, these new features in the XRD pattern are indeed signature for dynamical lattice distortion, which can be clearly observed in the experimental XRD spectrum as well (most notably, the ones within 20° and 30°). The good agreement between theory and experiment demonstrates again the presence of symmetry breaking induced by dynamical octahedral tilting in pseudocubic CsPbI_3_.

### 2.3 Impact of Dynamical Lattice Distortion on Band Structure

**Figure 3:**
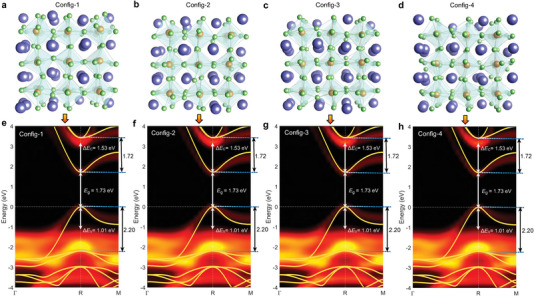
Electronic band structure of CsPbI_3_ at room temperature. a–d) Atomic structures of the four representative configurations from the AIMD trajectory. e–h) The corresponding unfolded band structures of the four selected configurations at room temperature. The yellow lines depict the band structure of cubic CsPbI_3_ at 0 K. The experimental values of Δ*E*_*c*_ and Δ*E*_*v*_ are provided in the plots for comparison and the intra‐VB and intra‐CB transition energies of cubic CsPbI_3_ are marked with black arrows.

To quantitatively study the impact of dynamical lattice distortion on the electronic structure, we select four representative configurations from the AIMD trajectory. The four configurations are randomly sampled from four time intervals (1000−2000 fs, 2000−3000 fs, 3000−4000 fs, and 4000−5000 fs), with a criterion that their bandgaps at R are close to the averaged value after equilibration. **Figure** **3**a–d shows their atomic structures; these structures retain the overall cubic shape, but consistently exhibit severe distortions characterized by Pb‐I octahedral tilting. Then the key question is how would these structural distortions modulate the electronic structure.

The local octahedral tilting induces lattice distortions and also breaks the cubic symmetry of the lattice. Hence, to better present and analyze the electronic structure of the pseudocubic CsPbI_3_, we compute the band structures of the four representative configurations using accurate HSE+SOC scheme and unfold the band structure to the cubic Brillouin zone of the unit cell.^[^ ^30^ ^]^ The corresponding unfolded band structures are shown in Figure 3e–h. Three important insights can be extracted from these results.

First, there exists substantial band renormalization for the distorted configurations. As compared to the band structure of the ideal cubic CsPbI_3_ (yellow lines), the renormalized band structure shows drastically reduced intra‐VB and intra‐CB transition energies. In particular, Δ*E*_*v*_ decreases by about one half. The reduced transition energies now actually agree very well with the experimentally determined values, demonstrating that the local structural distortions are responsible for the discrepancy between theory and experiment observed above.

Second, for both the lowest CB and the highest VB the dispersions are severely altered, leading to heavier effective masses for both electrons and holes. The electron effective mass is enhanced by 65% (0.119 *m*_0_ vs 0.196 *m*_0_), and the hole effective mass increases by 88% (0.119 *m*_0_ vs 0.224 *m*_0_). We note that to reproduce the experimental bandgap of 1.73 eV, we used an HSE mixing of 0.53 for the pseudocubic structure at 300 K, which is smaller than the one used for the ideal cubic structure (0.87). However, as shown in Figure S2 and Table S1 (Supporting Information), if we use a consistent HSE mixing of 0.53 for the ideal cubic structure, the electron and hole effective masses are even smaller (0.102 *m*_0_ for electrons and 0.100 *m*_0_ for holes). The corresponding intra‐VB and intra‐CB transition energies become 1.78 and 1.64 eV, respectively, which are still much larger than the experimentally measured Δ*E*_*v*_ (1.01 eV) and Δ*E*_*c*_ (1.53 eV). This comparison demonstrates that the giant band renormalization is not caused by the large HSE mixing used for the ideal cubic structure.

Third, despite the fact that the four representative configurations have different local atomic structures, their electronic band structures are extremely similar. This implies that there is a weak dependence of the band structure on the configuration chosen in the AIMD simulations, once dynamical equilibrium has reached. The consistency between the unfolded band structures also means that our computed band structures well represent the electronic structure of distorted CsPbI_3_ at room temperature. We note that even for configurations with bandgaps slightly different from the averaged value after equilibration, their band structures are also very similar to the ones in Figure 3e–h.

Dynamical lattice distortion also affects the localization of wavefunctions. As shown in Figure S4 (Supporting Information), the initial wavefunctions in the cubic CsPbI_3_ display regular and uniform patterns at the VBM and CBM. As the simulation proceeds, partial localization of wavefunctions due to lattice distortion becomes apparent. However, the induced quantum wells from lattice distortion are shallow, resulting in relatively weak wavefunction localization.

**Figure 4:**
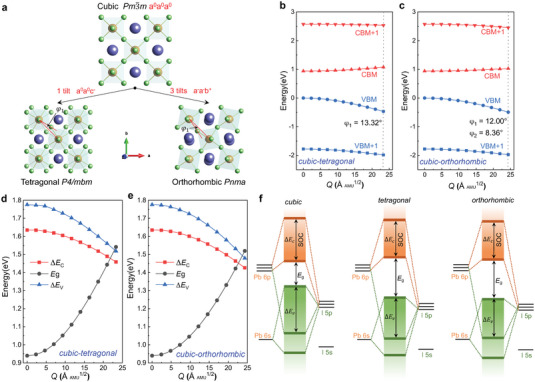
Impact of collective octahedral tilting on the electronic structure of CsPbI_3_. a) Structural transition of CsPbI_3_ from the cubic phase to the tetragonal or orthorhombic phases through octahedral tilting. b,c) Variation of the energy levels as a function of a generalized configurational coordinate (*Q*) during b) the cubic‐to‐tetragonal and c) cubic‐to‐orthorhombic phase transitions. The dotted lines indicate the location of the tetragonal or orthorhombic phase; the tilting angles of the Pb‐I octahedra are given. The VBM energy of cubic CsPbI_3_ is chosen as the zero reference. (d,e) Variation of the bandgap (*E*_g_), Δ*E*_*c*_, and Δ*E*_*v*_ during the d) cubic‐to‐tetragonal and e) cubic‐to‐orthorhombic transitions. f) Schematic orbital diagram of the cubic, tetragonal and orthorhombic phases of CsPbI_3_.

We further investigate the correlation between structural distortion and band renormalization in order to understand the microscopic mechanisms. As mentioned above, the structural distortions are primarily characterized by local dynamical tilting of the Pb‐I octahedra. The octahedral tilting is well‐known for perovskites, and is also a key ingredient of the structural transitions from the cubic phase to the tetragonal or orthorhombic phases.^[^ ^31^ ^]^ As shown in **Figure** **4**a, the Pb‐I octahedra only need to collectively rotate around the *c*‐axis in order to transition from the cubic phase ($Pm\bar{3}m$) to the tetragonal (*P4/mbm*) phase (marked 1 tilt, a^0^a^0^c^−^, following the Glazer notation^[^ ^32^ ^]^); there is a rotation angle φ_1_. For the transition between the cubic and orthorhombic (*Pnma*) phases, the Pb‐I octahedra rotate around three axes, which is labeled 3 tilts, a^−^a^−^b^+^. There are two distinct rotation angles: φ_1_ (in‐plane) and φ_2_ (out‐of‐plane). It is difficult to examine the tilting of a single octahedron and to take into account the dynamical disordering effect. Hence, we instead analyze the impact of collective octahedral tilting on the electronic structure during the cubic‐to‐tetragonal and cubic‐to‐orthorhombic phase transitions; the fundamental mechanisms are the same. For this analysis, we constrain the overall cubic shape, but allow Pb‐I octahedra to tilt in order to transition from the cubic phase to the “tetragonal” or “orthorhombic” phases. The rotation angle for the cubic‐to‐tetragonal transition is 13.32° (φ_1_), while the two rotation angles for the cubic‐to‐orthorhombic transition are 12.00° (φ_1_) and 8.36° (φ_2_).

We analyze the variation of the energies as a function of a generalized configuration coordinate (*Q*) that characterizes the structural transformation from the cubic to tetragonal or orthorhombic phases. *Q* is defined as the atomic mass (*m*_*i*_) weighted Cartesian coordinate (**R**_*i*_) with respect to the reference configuration—cubic perovskite (**R**_0;*i*_), $Q=\sqrt{\sum_{i}m_{i}{\left(R_{i}-R_{0;i}\right)}^{2}}$.^[^ ^27^ ^]^ As depicted in Figure 4b, with the increase in the structural distortion (i.e., the octahedral tilting angle) during the cubic‐to‐tetragonal transition the VBM level shows a pronounced decrease and the CBM level slightly increases. The CBM+1 energy remains almost unchanged, while the VBM+1 energy gradually deceases with increasing distortion; but the decrease in the VBM+1 energy is less pronounced than that in the VBM. Similar behavior is also found for the cubic‐to‐orthorhombic phase transition (Figure 4c).

Figure 4d,e plot variation of the transition energies (*E*_g_, Δ*E*_*c*_, and Δ*E*_*v*_) in CsPbI_3_ during the cubic‐to‐tetragonal and cubic‐to‐orthorhombic phase transitions. Obviously, *E*_g_ gradually increases, similar to the trend as observed in the first 1000 steps of the AIMD simulations. Both Δ*E*_*c*_ and Δ*E*_*v*_ decrease rapidly with increasing the octahedral tilting. Our detailed analysis of the atomic structures in Figure 3a–d shows that the local rotation angle of Pb‐I octahedra can reach up to 24.35°, which is 2‐3 times larger than the ones associated with the cubic‐to‐tetragonal and cubic‐to‐orthorhombic transitions. Also, in pseudocubic CsPbI_3_ at room temperature there exists octahedral tilting in three principal axes. The large tilting of Pb‐I octahedra leads to substantial reduction of Δ*E*_*c*_ and Δ*E*_*v*_, which explains the giant band renormalization for the pseudocubic CsPbI_3_ at room temperature observed in our first‐principles calculations (Figure 3e–h) and the large discrepancy between theory and experiment (Figure 1a,b).

The variations of the VBM and CBM energies and transition energies can be understood by analyzing the orbital diagram of CsPbI_3_ in three different phases. As schematically shown in Figure 4f, the VBM of CsPbI_3_ perovskites is an antibonding state between Pb 6*s* and I 5*p* orbitals, and the VBM+1 is a bonding state between the Pb 6*p* and I 5*p* orbitals.^[^ ^33^ ^]^ Both the CBM and CBM+1 are antibonding states of Pb 6*p* and I 5*p* orbitals,^[^ ^34^ ^]^ but they are split due to strong SOC and local crystal field. As the octahedron tilts, the local symmetry is reduced, leading to reduced *s*‐*p* coupling (between VBM and lower‐lying Pb 6*s* states) and enhanced *p*‐*p* coupling (between CBM/CBM+1 and VBM/VBM+1).^[^ ^35^ ^]^ The enhanced *p*‐*p* coupling increases the CBM and CBM+1 energies, while decreases the VBM and VBM+1 energies. The reduced *s*‐*p* coupling does not affect the CBM and VBM+1 energies, but further lowers the VBM energy. The octahedral tilting also increases the Pb‐I bond length, which, as a secondary effect, decreases the CBM and VBM energies, and slightly increases the VBM+1 energy. Consequently, the energy difference between the VBM and VBM+1 (Δ*E*_*v*_) decreases. As discussed above, the CBM+1 energy should, in principle, increase due to enhanced *p*‐*p* coupling. Nevertheless, while the octahedron rotates, the *p*_*x*_, *p*_*y*_, and *p*_*z*_ orbitals start to mix, which results in an effective lowering of the SOC splitting. Hence, the CBM+1 energy slightly decreases, and the energy difference between CBM and CBM+1 (Δ*E*_*c*_) is also reduced.

### 2.4 Implications for Carrier Dynamics

The giant band renormalization has direct relevance to the carrier dynamics in lead‐iodide perovskites. The most straightforward consequence is that the carrier transport would be much slower. As we discussed before, the giant band renormalization leads to increases in both the electron and hole effective masses by 65% and 88%, respectively. The band dispersions as characterized by the carrier effective masses directly influence the carrier mobilities. Within the deformation potential theory, the carrier mobility (μ) relates to the effective mass (m_*_) by^[^ ^36^ ^]^

(1) $$\mu =\frac{2\sqrt{2\pi }e\hbar^{4}c_{ii}}{3m_{*}^{5/2}{\left(k_{B}T\right)}^{3/2}E_{1}^{2}},$$

where *e* is elementary charge, ℏ the Planck constant, *c*_*ii*_ the elastic constant, *k*_B_ the Boltzmann constant, *T* the absolute temperature, and *E*_1_ a deformation‐potential constant. Based on Equation (1), one can easily estimate that a 65% (88%) increase in the electron (hole) effective mass would decrease the electron (hole) mobility by 72% (89%), which is a very pronounced effect and would substantially impact the device performance. Actually, previous first‐principles calculations^[^ ^37^ ^]^ have found that the explicitly computed carrier mobilities for cubic CsPbI_3_ are higher than the experimental values^[^ ^38^, ^39^ ^]^ by around one order of magnitude. While there are many factors that could be responsible for the large difference, the giant band renormalization might be a relevant mechanism. The suppressed carrier transport by dynamical lattice distortion might also provide an intuitive explanation to the unexpectedly low carrier mobilities in halide perovskites observed in experiments.^[^ ^40^ ^]^ With the identification of a possible cause of low carrier mobilities, effective strategies might be developed to improve carrier transport in halide perovskites via lattice and band‐structure engineering.

Further, the band renormalization pushes the VBM+1‐to‐CBM transition from the ultraviolet to the visible spectral region (see Figure 3e–h), and hence increases the material absorption in the visible. Although the absorption coefficients of these halide perovskites are often sufficiently large, this enhancement might still be important when the light‐absorption layer has a limited thickness.

**Figure 5:**
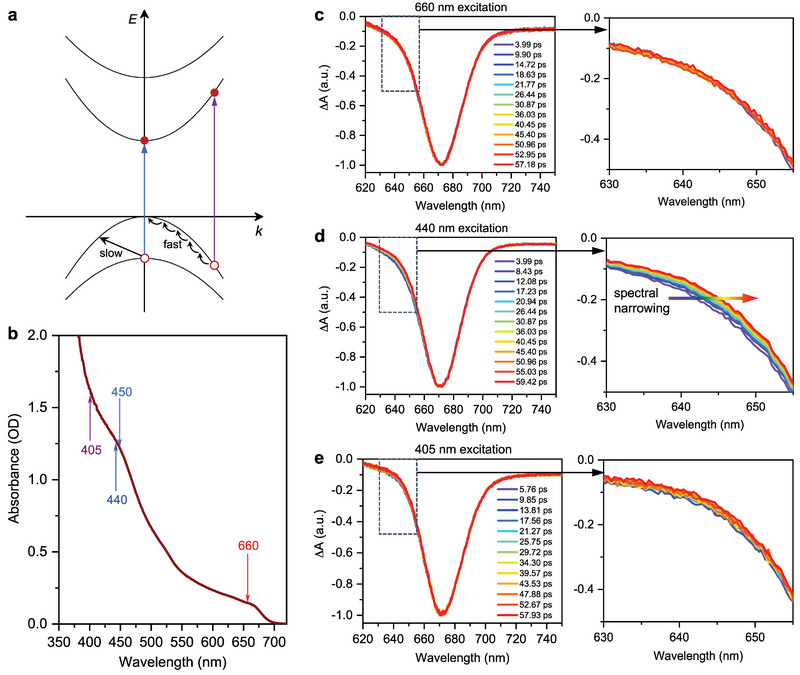
Slow hot‐hole cooling enabled by band renormalization. a) Schematic band structure and hot‐hole relaxation in lattice‐distorted CsPbI_3_. Under 405 nm excitation (purple arrow), the high‐*k* hot hole can rapidly relax by emitting phonons; under 440 nm excitation (blue arrow), energy‐momentum mismatch might result in relatively slow hot‐hole relaxation between the two valence bands. b) Absorption spectrum of the CsPbI_3_ nanocrystals (brown solid line) and the different pump wavelengths (colored arrows) used to observe hot‐carrier cooling. c–e) Time‐dependent transient absorption spectra normalized at their bleach maxima under c) 660 nm, d) 440 nm, and e) 405 nm excitations. The highlighted regions in the left panels are enlarged in the right panels. A progressive spectral narrowing on the blue side of the bleach is absent in (c), particularly obvious in (d), and less as obvious in (e).

As to the dynamical aspect, the strong reduction of the intra‐VB transition energy (Δ*E*_*v*_) might significantly alter the hot‐carrier relaxation dynamics. In the scenario of cubic CsPbI_3_, blue‐light excitation creates highly‐energetic carriers at high‐*k* states in the lowest CB and highest VB. These hot carriers can rapidly relax by emitting phonons. Now with the distorted CsPbI_3_, blue‐light excitation generates hot holes in the dispersion curve of VBM+1 (see **Figure** **5**a). If the energy‐momentum mismatch between VBM+1 and the highest VB is not easily accommodated by phonons, there might be long‐sustained hot holes in VBM+1, which could be harvested for efficient hot‐carrier devices. We design pump wavelength‐dependent TA experiments to verify this hypothesis. As plotted in Figure 5b, we adopt four different pump wavelengths, among that 660 nm is slightly above the VBM‐to‐CBM transition; 450 and 440 nm are in resonance with the VBM+1‐to‐CBM transition, whereas 405 nm is off resonance with the VBM+1‐to‐CBM transition and mostly contributed by high‐*k* VB‐to‐CB transitions.

The broadband TA spectra are presented in Figure S5 (Supporting Information). All the spectra are controlled to have approximately identical signal sizes in order to ensure a similar level of carrier occupancies with different pump wavelengths. The major distinction between 660 nm and other shorter wavelength excitations lies in the first few ps. Short‐wavelength excitations result in state‐filling bleaches and photoinduced absorption features on the blue and red sides, respectively, of the lowest bleach peak (Figure S5, Supporting Information), a manifestation of hot‐carrier population.^[^ ^41^, ^42^ ^]^ These features disappear within ∼2 ps, indicating that the majority of the hot carriers relax to CBM and VBM within this time. This ultrafast hot‐carrier cooling is a typical observation that has been extensively studied in previous studies. Therefore, we will not elaborate on this here. Instead, here we focus on a minor, slow part of the hot‐carrier relaxation. In principle, we should be able to directly track hot‐carrier dynamics by probing the B2 feature at ca. 420 nm. However, strong absorptions of the sample near this region result in strong attenuation of the probe light and hence very poor (or even undetectable) transient absorption signals near B2. Further, as shown in Figure 1b, the B2 feature is present even if we pump resonantly into the B1 feature, as they share the common CBM level, that is, the B2 feature contains significant contribution from band‐edge carriers. These two issues, in combination with the very low portion of long‐lived hot carriers, prohibit direct measurement of hot‐hole relaxation by following the B2 kinetics. Instead, we choose to monitor the carrier dynamics probed near the band edge where the signal/noise ratio of the transient absorption signals is optimal. Specifically, if we normalize the TA spectra from ∼4 to 60 ps to their bleach maxima, we find that the spectra still differ for different pump wavelengths. The 660 nm excited spectra have essentially no spectral evolution on this timescale (Figure 5c), as expected for the absence of hot‐carrier relaxation under this pump wavelength. By contrast, the 440 nm excited spectra are well normalized to the red side but show progressive spectral narrowing on the blue side (Figure 5d), which can be better seen in the enlarged view to the right. This is a fairly reproducible phenomenon that can be observed with 450 nm excitation as well (Figure S6, Supporting Information). This spectral narrowing is indicative of a minor but still discernible portion of slow hot‐carrier relaxation.^[^ ^43^, ^44^, ^45^ ^]^ Most importantly, under 405 nm excitation, the spectral narrowing on the blue side is less prominent than the 440 nm case (Figure 5e). In principle, under a similar level of carrier occupancies, one should expect a slower hot‐carrier relaxation for 405 nm excitation, as in this case the carriers simply have more energies to dissipate.

The counterintuitive behavior, however, can be well rationalized by invoking the VBM+1‐to‐CBM transition mentioned above. Because 440 and 450 nm are in resonance with this transition, they excite hot holes into VBM+1 and these hot‐holes display slow relaxation likely due to the energy‐momentum mismatch between VBM+1 and the highest VB speculated above (Figure 5a), that is, the energy and momentum released in this transition cannot be simultaneously accommodated by one phonon (most likely the longitudinal optical phonon). Because hot carriers relax in a cascade‐like fashion, this “bottleneck” also slows down the ensuing relaxation events, eventually being manifested as slowly‐decaying blue‐side bleaches in Figure 5d and Figure S6 (Supporting Information). By contrast, 405 nm excitation is off resonance with the VBM+1‐to‐CBM transition and becomes dominated by high‐*k* VB‐to‐CB transitions; the resulting hot carriers can rapidly relax by emitting phonons.

While we cannot exclude the existence of such a hot‐hole relaxation “bottleneck” in an ideal cubic CsPbI_3_, the key is that lattice distortion has brought this effect into a solar‐relevant regime (near 440 nm excitation). Otherwise, according to our calculation, in the ideal cubic structure this transition will be situated at >3.9 eV (Figure 1b), which is hard to access by solar photons. Although the contribution of the VBM+1‐to‐CBM transition to the overall optical absorbance in the range remains small, and therefore the slow hot‐hole relaxation has a small amplitude, it is still a very promising observation as it opens a new avenue of engineering hot‐carrier relaxation dynamics through lattice distortion and band renormalization in halide perovskites. It also offers an alternate explanation to the slow hot‐carrier relaxation dynamics observed in these materials that have been generally explained using a polaronic screening mechanism.^[^ ^46^ ^]^

Besides hot‐carrier cooling, the strong band renormalization might also have important consequences for three‐carrier Auger recombination that involves excitation of a carrier into high‐energy states.^[^ ^21^, ^47^, ^48^ ^]^ The structure of high‐energy bands dictates the transition momentum and density of the final states, and hence controls the rate of Auger recombination. In a similar way, the structure of high‐energy bands could influence the “inverse” process of Auger recombination—carrier multiplication,^[^ ^47^, ^49^, ^50^ ^]^ a process by which the photovoltaic efficiency can be strongly enhanced. Verification of these consequences is considerably more complex than hot‐carrier cooling, but certainly warrants future efforts. Overall, many possibilities might be opened by the lattice distortion and strong band renormalization in halide perovskites.

## 3 Conclusion

To conclude, we have unveiled a giant electronic band renormalization in pseudocubic halide perovskite CsPbI_3_ due to dynamical lattice distortion in the form of octahedral tilting, which has been commonly overlooked. The electronic band renormalization critically influences the carrier dynamics, which enables a new pathway to engineer carrier transport and relaxation via lattice distortion. This conclusion is expected to be applicable to both all‐inorganic and organic‐inorganic hybrid perovskites (Figure S7, Supporting Information). We call for a reassessment of the previously computed electronic structures of halide perovskites, and properties that sensitively depend the electronic band structure such as effective masses, absorption, luminescence, carrier mobilities, and carrier recombination rates.

## 4 Experimental Section

### First‐Principles Calculations

All first‐principles calculations in this work were performed using the projector augmented‐wave (PAW)^[^ ^51^ ^]^ pseudopotentials as implemented in the Vienna ab initio simulation package (VASP). The experimental lattice constant of 6.17 Å for cubic CsPbI_3_ was adopted.^[^ ^25^ ^]^ The HSE^[^ ^14^ ^]^ hybrid functional including the SOC effect was employed for the first‐principles calculations. A mixing parameter of 0.87 for the Fock exchange was used for the ideal cubic CsPbI_3_ in order to reproduce the experimental bandgap, 1.73 eV.^[^ ^25^ ^]^ The plane‐wave energy cutoff was set to 500 eV and and a 6 ×6 ×6 Monkhorst–Pack^[^ ^52^ ^]^ **k**‐point grid was adopted for the cubic unit cell of CsPbI_3_.

A 3× 3×3 supercell of cubic CsPbI_3_ containing 135 atoms was used for the AIMD simulations. For computational affordability, the AIMD simulations were carried out using the functional by Perdew, Burke, and Ernzerhof (PBE),^[^ ^53^ ^]^ and SOC was not included. A canonical ensemble (NVT) with the Nosé‐Hoover thermostat^[^ ^54^, ^55^ ^]^ at 300 K was employed. The time step for the AIMD simulations was 1 fs. The PBE+SOC scheme was utilized to calculate the partial charge density at the R point for selected configurations from the AIMD trajectory. A time step of 0.5 ps was employed to capture the dynamic changes in the wavefunction. This approach allowed to effectively visualize the spatial variations of the wavefunction throughout the simulation. A mixing parameter of 0.53 within the HSE+SOC scheme was used for the supercell band‐structure calculations, which reproduces the experimental bandgap after unfolding. For unfolding the electronic band structures of disordered supercells, the scheme implemented in VASPKIT was employed.^[^ ^56^ ^]^

To simulate the cubic‐to‐tetragonal and cubic‐to‐orthorhombic phase transitions, a $\sqrt{2}\times \sqrt{2}\times$2 supercell was built from the cubic unit cell. This study adopted the same plane‐wave energy cutoff and a 4 × 4 × 3 **k**‐point grid for the HSE+SOC calculations.

### Sample Synthesis and Characterizations

The black‐phase CsPbI_3_ nanocrystals were synthesized using a hot‐injection method reported in previous studies.^[^ ^48^ ^]^ For TEM characterizations, the nanocrystals in hexane were dropped onto ultrathin carbon TEM grids. The images were acquired on a JEOL JEM‐2100 at 200 kV accelerating voltage. For XRD measurements, the nanocrystals were dried into solid powders and imported to a X‐ray Diffractometer (Empyrean) with a temperature‐controlled cell (TTK 450, Anton Paar GmbH). The diffraction patterns were acquired using Cu K_α_ radiation operated at 40 kV and 40 mA, with a step width of 0.026° in the 2θ range from 10° to 50°.

### Transient Absorption Spectroscopy

TA measurements were performed on two femtosecond‐amplifier‐based transient spectrometers. For broadband TA covering ∼350−800 nm range, a regenerative amplified Ti:sapphire laser system (800 nm, 1 kHz, 70 fs pulse‐duration; Coherent) and a TOPAS Optical Parametric Amplifier (OPA) were used. For hot‐carrier relaxation measurements, a Pharos laser (1030 nm, 100 kHz, 230 fs pulse‐duration; Light Conversion) and Orpheus‐HP OPA were used. The details were provided in previous studies.^[^ ^20^, ^48^ ^]^ It is noted that directly monitoring hot‐carrier relaxation at short wavelengths in TA spectroscopy is challenging due to the strong absorption of the current sample in that region.

## Conflict of Interest

The authors declare no conflict of interest.

## Supporting information

Supporting Information

## Data Availability

The data that support the findings of this study are available from the corresponding author upon reasonable request.
