# Supplementary material for: Engineering Carrier Dynamics in Halide Perovskites by Dynamical Lattice Distortion
Source: Adv Sci (Weinh). 2023 Oct 9;10(33):2300386. doi: 10.1002/advs.202300386 (PMC10667814; doi:10.1002/advs.202300386)
Supplement: Supplementary file 1 — Supporting Information [file ADVS-10-2300386-s001.pdf]

ADVANCED SCIENCE
Open Access

Supporting Information

for *Adv. Sci.*, DOI 10.1002/advs.202300386

Engineering Carrier Dynamics in Halide Perovskites by Dynamical Lattice Distortion

Bai-Qing Zhao, Yulu Li, Xuan-Yan Chen, Yaoyao Han, Su-Huai Wei, Kaifeng Wu* and Xie Zhang**

Supporting Information for "Engineering carrier dynamics in halide perovskites by dynamical lattice distortion"

Bai-Qing Zhao[#], Yulu Li[#], Xuan-Yan Chen, Yaoyao Han, Su-Huai Wei*, Kaifeng Wu*, and Xie Zhang*

[#]*Bai-Qing Zhao and Yulu Li contributed equally.*

Bai-Qing Zhao, Xuan-Yan Chen, Prof. Su-Huai Wei
Beijing Computational Science Research Center, Beijing 100193, China
Email Address: suhuaiwei@csrc.ac.cn
Yulu Li
State Key Laboratory of Molecular Reaction Dynamics, Dalian Institute of Chemical Physics, Chinese Academy of Sciences, Dalian, Liaoning 116023, China
Yaoyao Han, Kaifeng Wu
State Key Laboratory of Molecular Reaction Dynamics, Dalian Institute of Chemical Physics, Chinese Academy of Sciences, Dalian, Liaoning 116023, China
University of Chinese Academy of Sciences, Beijing 100049, China
Email Address: kwu@dicp.ac.cn
Prof. Xie Zhang
School of Materials Science and Engineering, Northwestern Polytechnical University, Xi'an 710072, China
Email Address: xie.zhang@nwpu.edu.cn

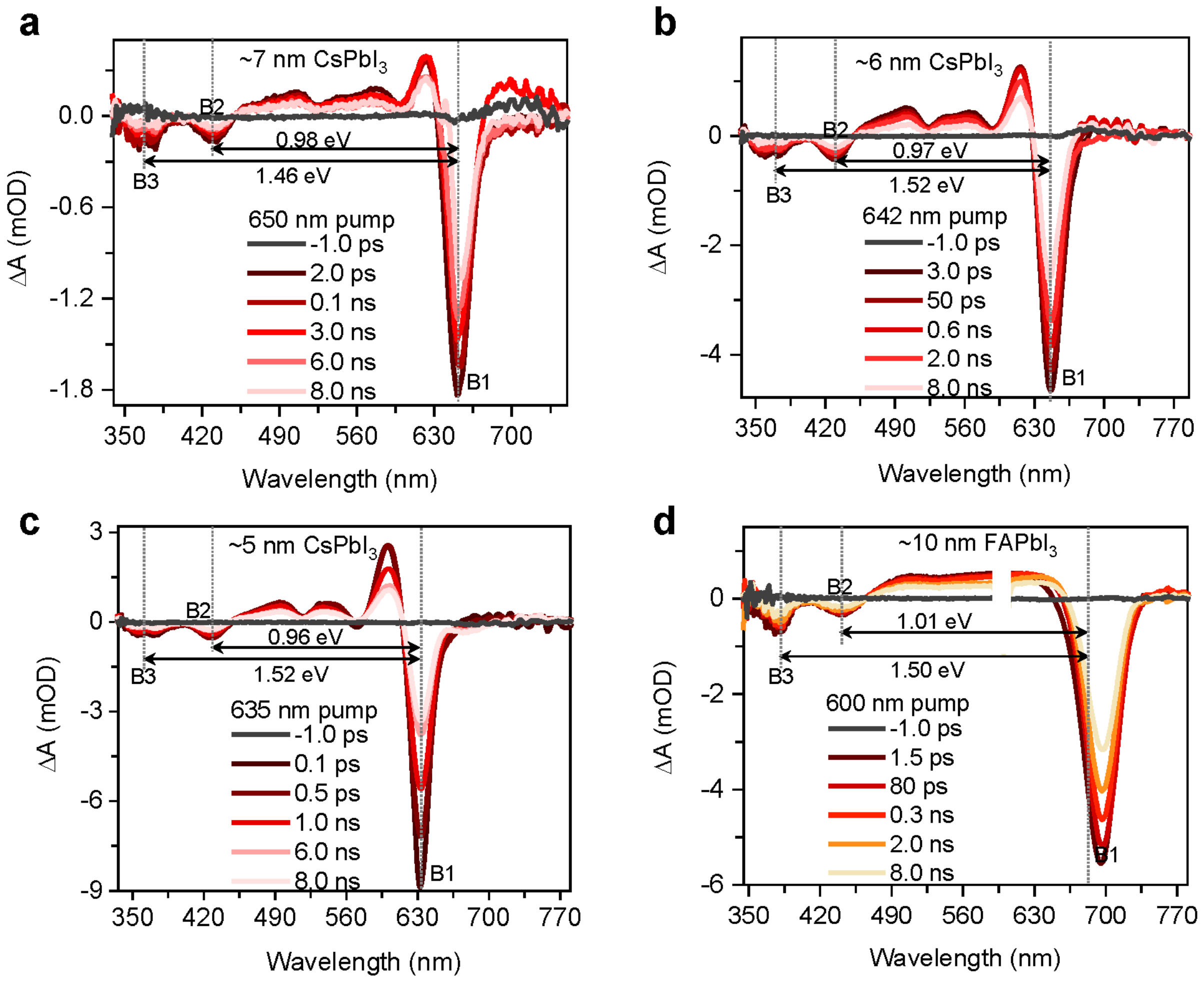

Figure S1. Additional transient absorption (TA) spectra. TA spectra of (a) ~7 nm, (b) ~6 nm and (c) ~5 nm $CsPbI_3$ nanocrystals and (d) $FAPbI_3$ (FA: formamidinium) nanocrystals at varying probe delays following excitation using the indicated pump wavelengths. The three bleach features, B1, B2, and B3, and the energy separation between them, are labeled. The $CsPbI_3$ nanocrystals sizes (edge lengths) are reported in prior studies[1,2].

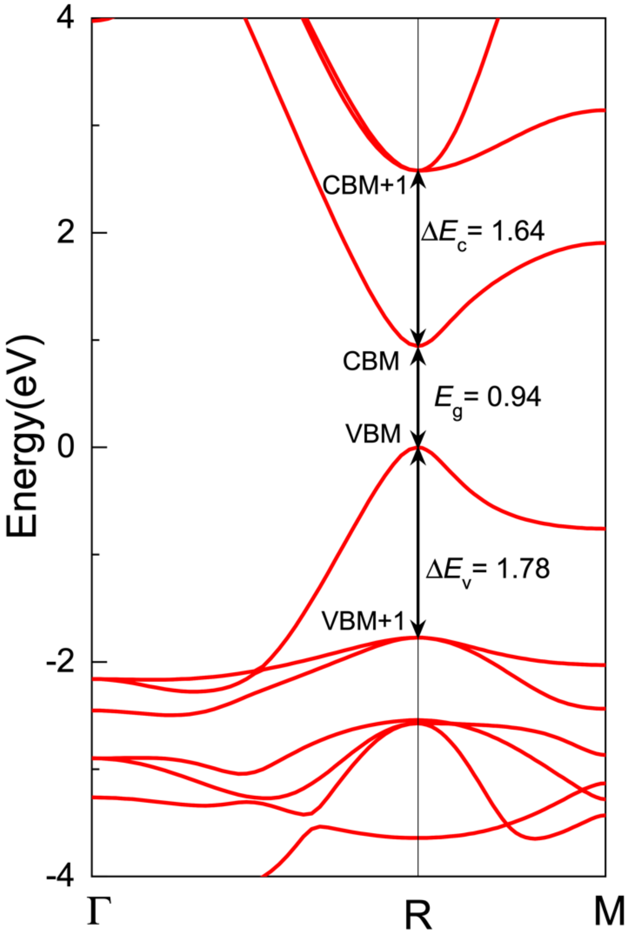

Figure S2. Band structure of cubic $CsPbI_3$ computed from the HSE hybrid functional with a mixing parameter of 0.53.

Table S1. Computed properties for cubic and lattice-distorted cubic $CsPbI_3$ (at 300 K) from first principles with different HSE mixing parameter α.

Structure	E_g (eV)	ΔE_c (eV)	ΔE_v (eV)	m_e (m_0)	m_h (m_0)
Cubic ($\alpha = 0.87$)	1.73	1.72	2.20	0.119	0.119
Cubic ($\alpha = 0.53$)	0.94	1.64	1.78	0.102	0.100
Distorted cubic@300 K ($\alpha = 0.53$)	1.73	1.53	1.01	0.196	0.224

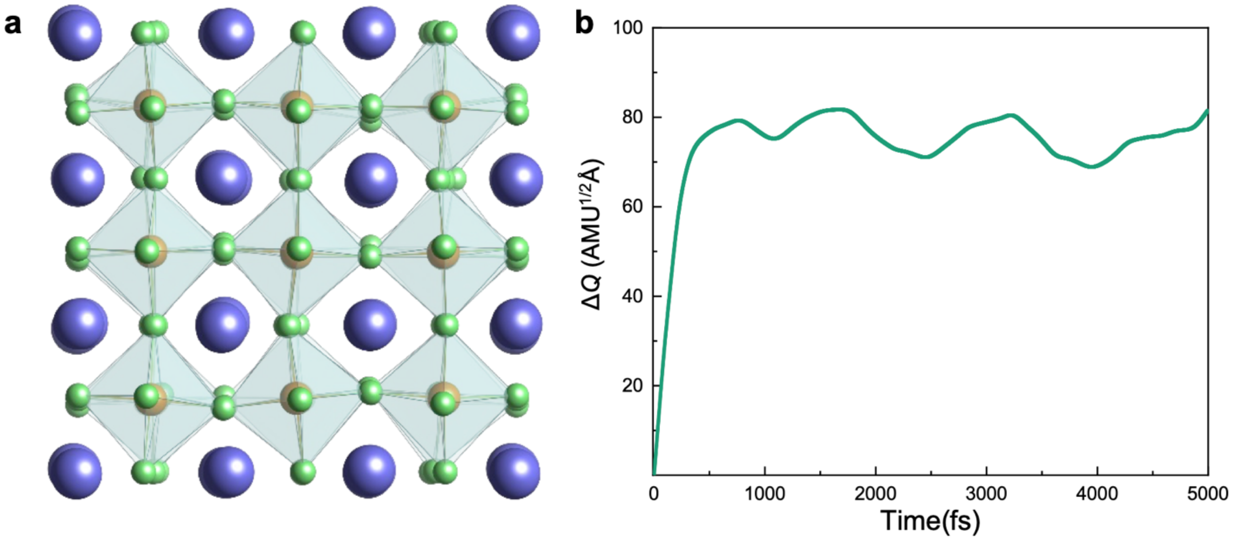

Figure S3. (a) Ensemble-averaged atomic structure of $CsPbI_3$ from the AIMD simulations. (b) Structural difference (ΔQ) of AIMD configurations from the ideal cubic perovskite as a function of the simulation time.

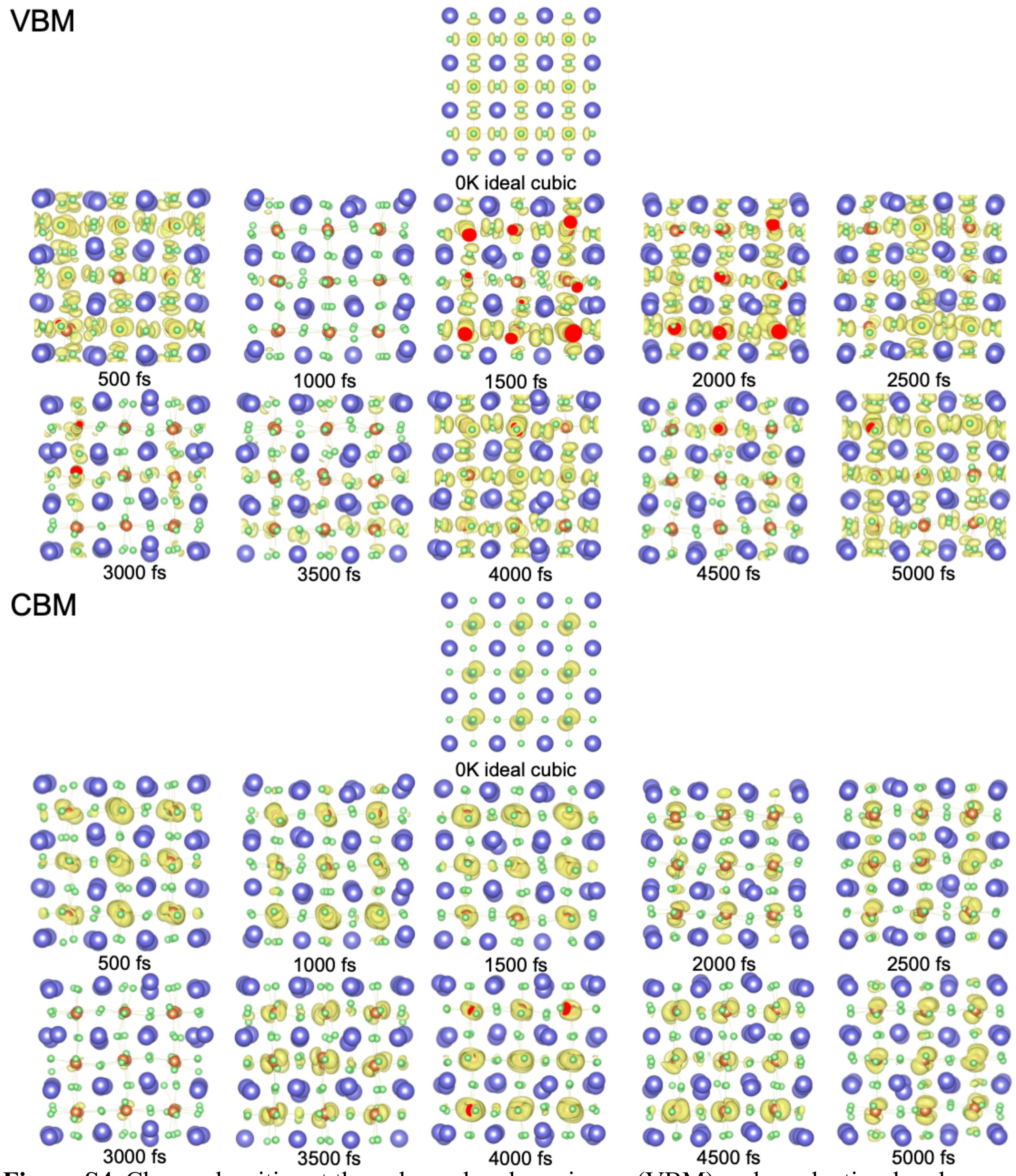

Figure S4. Charge densities at the valence-band maximum (VBM) and conduction-band minimum (CBM) with a time step of 0.5 ps in the AIMD simulation.

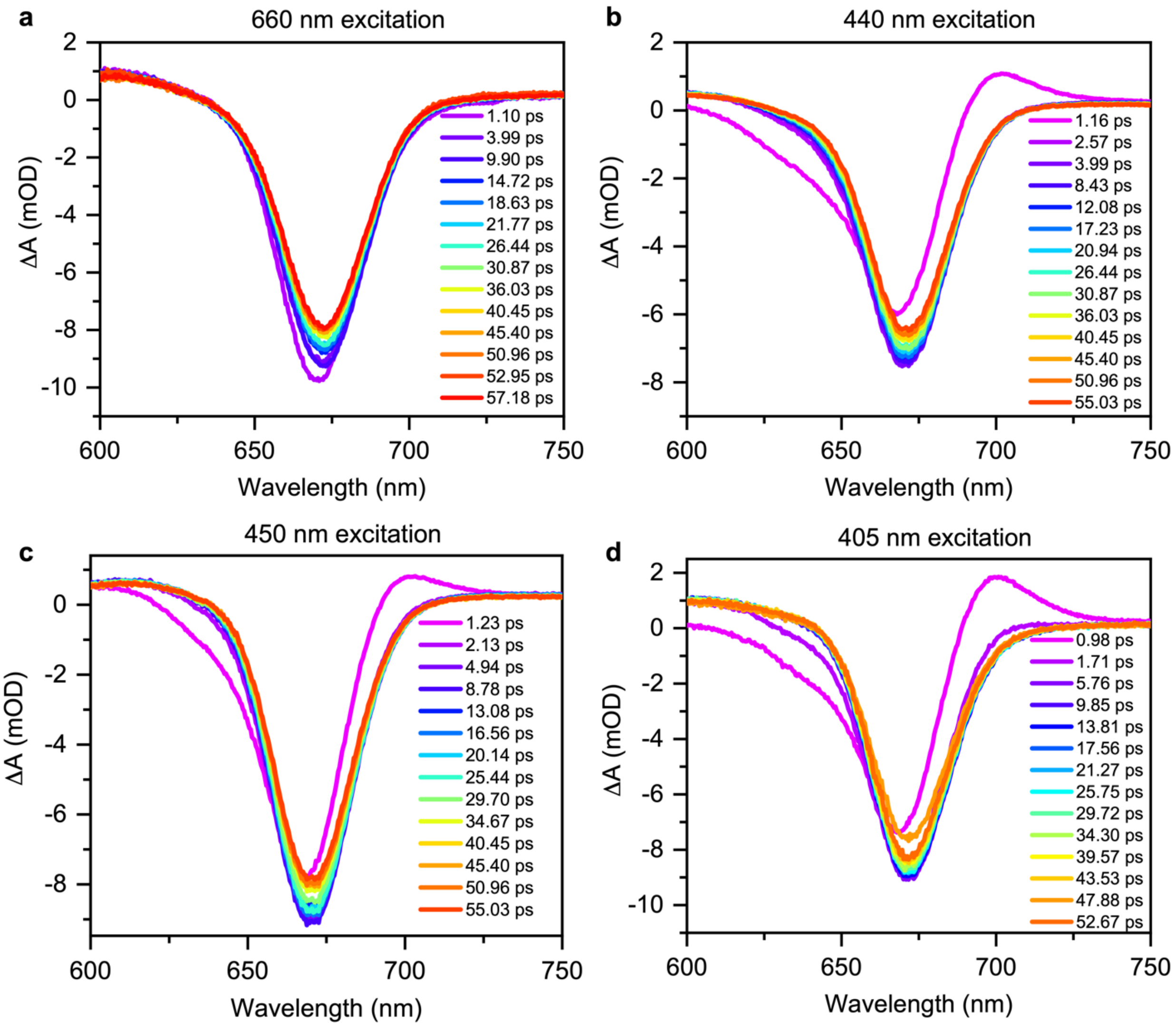

Figure S5. TA spectra of $CsPbI_3$ nanocrystals under different pump wavelengths. TA spectra at varying probe delays following excitation at (a) 660 nm, (b) 450 nm, (c) 440 nm, and (d) 405 nm. Note that these spectra are original spectra whereas those presented in Figure 5 and Figure S5 below are normalized to their spectral bleach maxima.

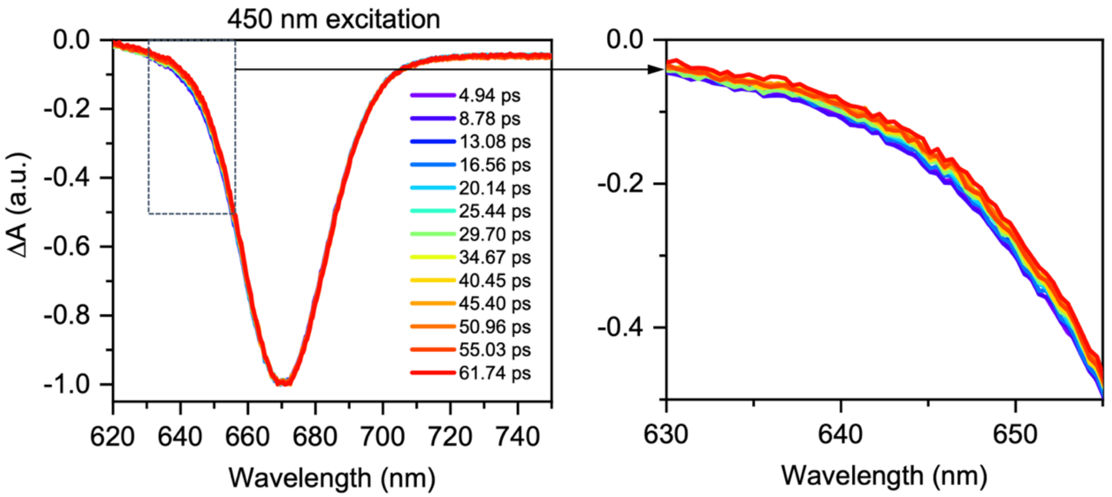

Figure S6. Hot-carrier cooling under 450 nm excitation. Time-dependent TA spectra normalized at their bleach maxima. The highlighted regions in left panels are zoomed-in in the right panels.

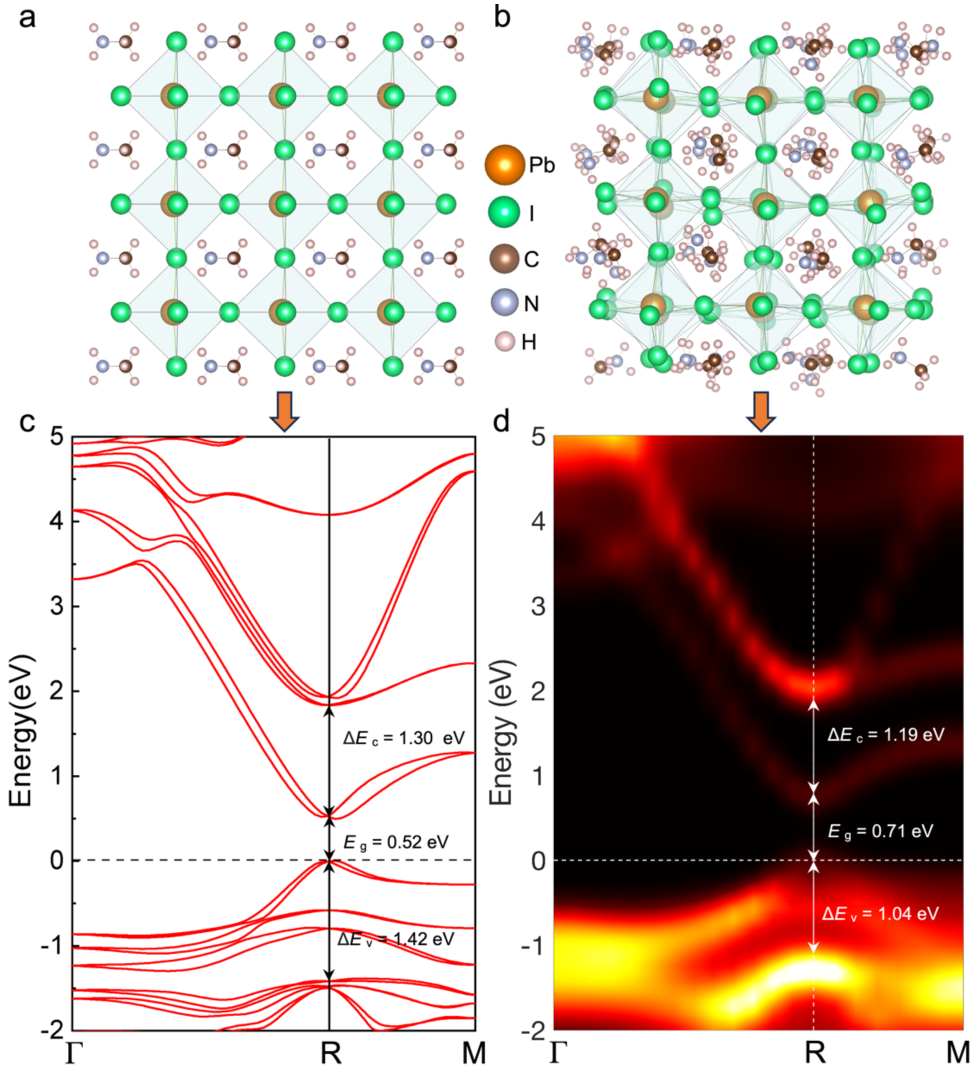

Figure S7. Electronic band structure of $MAPbI_3$. (a,b) Atomic structures of $MAPbI_3$ at (a) T = 0 K and (b) T = 300 K (obtained from *ab initio* molecular dynamics simulations at room temperature). (c,d) The corresponding electronic band structures for the atomic structures at (c) T = 0 K and (d) T = 300 K, using the Perdew-Berke-Ernzerhof (PBE) functional in conjunction with spin-orbit coupling (SOC).

Supplementary references

1 Li, Y., Luo, X., Liu, Y., Lu, X. & Wu, K. Size- and Composition-Dependent Exciton Spin Relaxation in Lead Halide Perovskite Quantum Dots. *ACS Energy Lett.* **5**, 1701-1708 (2020).

2 Li, Y., Luo, X., Ding, T., Lu, X. & Wu, K. Size- and Halide-Dependent Auger Recombination in Lead Halide Perovskite Nanocrystals. *Angew. Chem. Int. Ed.* **59**, 14292-14295 (2020).
